# Transradial versus Transfemoral Access and the Risk of Acute Kidney Injury following Primary Percutaneous Coronary Intervention in Patients with ST-Elevation Myocardial Infarction: A Systematic Review and Meta-Analysis of Randomized Controlled Trials and Propensity-Score-Matched Studies

**DOI:** 10.1155/2022/6774439

**Published:** 2022-03-10

**Authors:** Gaspar Del Rio-Pertuz, Michel Juarez, Poemlarp Mekraksakit, Kanak Parmar, Mohammad M. Ansari

**Affiliations:** ^1^Department of Internal Medicine, Texas Tech University Health Sciences Center, Lubbock, TX, USA; ^2^Center for Research in Indigenous Health, Wuq'Kawoq-Maya Health Alliance, Chimaltenango, Guatemala; ^3^Division of Cardiology, Texas Tech University Health Sciences Center, Lubbock, TX, USA

## Abstract

**Objectives:**

The aim of this study is to examine the association between vascular access sites and the incidence of AKI in patients with STEMI undergoing primary PCI.

**Background:**

Emerging evidence has suggested that transradial access (TRA) may be associated with lower rates of acute kidney injury (AKI) as compared with transfemoral access (TFA). However, most of these studies have included a nonselected study population undergoing diagnostic cardiac catheterization or percutaneous coronary intervention (PCI). Data on the association between TRA and AKI in this setting of STEMI are limited and with conflicting results.

**Methods:**

We systematically searched PubMed, Embase, and Scopus for abstracts and full-text articles from inception to July 13^th^ of 2021. Studies included were randomized controlled trials (RCTs) and propensity-score-matched (PSM) studies evaluating the association of TRA versus TFA access with AKI in patients undergoing primary PCI for STEMI. Data were integrated using the random effects model and generic inverse‐variance method of DerSimonian and Laird.

**Results:**

A total of 10,093 studies were found. After applying our inclusion criteria, 5 studies from 2014 to 2021 with a total of 8,536 STEMI patients were included. TRA was not significantly associated with a reduced risk for AKI compared with TFA (odds ratio 0.85, 95% CI 0.71–1.01, *p* 0.07, *I*^2^ = 40%).

**Conclusions:**

Transradial access was not significantly associated with lower risk of AKI in patients undergoing primary PCI for STEMI compared with TFA. Larger studies are needed to clarify this outcome.

## 1. Introduction

Primary percutaneous coronary intervention (PCI) is the recommended management approach for coronary reperfusion in patients presenting with ST-segment myocardial infarction (STEMI). Acute kidney injury (AKI) is one of the most common complications of acute coronary syndrome (ACS), and its presence after STEMI is a predictor of in-hospital and 1-year mortality [1]. Pathophysiology of AKI in patients undergoing either PCI or any coronary diagnostic procedure is multifactorial, involving contrast volume, impaired systemic and renal hemodynamic conditions, imbalance of endogenous vasodilating and vasoconstrictive factors, and direct cholesterol embolization [2–4].

Risk factors for AKI after PCI can be divided into reversible and irreversible factors. Irreversible factors are patient-dependent, such as age, presence of chronic comorbidities, for example, diabetes mellitus, preexisting renal impairment, history of renal transplantation, or the use of nephrotoxic medications. On the other hand, reversible factors are procedure-dependent such as presence of anemia before the procedure, contrast media properties, contrast media volume, number of procedures, and vascular access selected.

In recent years, there is a trend in the use of transradial access (TRA) over transfemoral access (TFA) when performing coronary angiography or PCI due to an association between fewer bleeding events and lower mortality with TRA [5, 6]. Emerging evidence suggested that TRA might be associated with lower rates of AKI as compared with TFA in patients undergoing either PCI or coronary angiography [7–9]. However, the results have been inconsistent, and most of these studies have included a nonselected study population of patients undergoing diagnostic cardiac catheterization or PCI.

Different randomized controlled trials (RCT) and observational studies evaluating the association between TRA and AKI in the setting of STEMI have been published but are limited with conflicting results, due to the limited evidence in support of access site as an independent contributor to renal dysfunction in STEMI. We, therefore, performed a detailed systematic review and meta-analysis to investigate the association between vascular access sites and the incidence of AKI in patients with STEMI undergoing primary PCI.

## 2. Method

### 2.1. Data Search

The present systematic review and meta-analysis was performed according to the Preferred Reporting Items for Systematic Reviews and Meta-Analyses (PRISMA) statement [10] and the guideline for meta-analysis of observational studies in epidemiology (MOOSE) [11]. For the purpose of the study, we systematically searched PubMed, Embase, and Scopus database from inception to July 13^th^ 2021. We also checked the reference for any included articles. The search strategy consisted of a combination and variation of the terms “radial,” “transradial,” “femoral,” “transfemoral,” “coronary angiography,” “percutaneous coronary intervention,” “left heart catheterization,” “ST-elevation myocardial infarction,” “STEMI,” “acute kidney injury,” and “contrast induced nephropathy.” The actual strategy listing all search terms used and how they are combined is available in Appendix. This study has been registered at PROSPERO International prospective register of systematic reviews under registration number: CRD42021268798.

### 2.2. Inclusion Criteria

The population, intervention, comparison, outcome, and study design model was used to select studies for this systematic review [10]. Selection criteria used to identify studies included human studies in English, RCT, and prospective or observational studies that were conducted on adult patients (>17 years old) with STEMI (population) requiring PCI. Studies were required to compare the radial rout (intervention) with the femoral route (comparison) for catheterization. The outcome of the study was required to be the incidence of AKI. The definition of AKI was as per the included study. Studies not reporting relevant data, studies reporting duplicate data, single‐arm studies, case series, case reports, non‐English language studies, and review articles were excluded.

### 2.3. Data Extraction

Title and abstracts of all articles retrieved using the search strategy were initially screened, reviewed, and verified independently by two authors (GDRP and MJ), with any disagreements mediated through discussion with a third review author (MMA). The full texts of potentially eligible articles were reviewed by (GDRP and MJ), with disagreements mediated by (MMA). For all included articles, a single preformatted abstraction form was used by two authors (GDRP and MJ) to extract the data; any discrepancy was mediated by a discussion with a third author (MMA). Details including the first author's name, publication year, sample size, demographic details, baseline characteristics of the study sample, definition of AKI, contrast volume, and incidence of AKI were extracted. The outcome extracted was the incidence of AKI. Odds ratio (OR) with 95% confidence intervals (CI) was abstracted or calculated based on the reported event rates. For studies reporting unadjusted, adjusted, or propensity score matched (PSM) data, the highest-quality estimate was picked for the overall meta-analysis using the following rank order: PSM > adjusted > unadjusted.

### 2.4. Statistical Analysis

We performed a meta-analysis of the included RCT and PSM studies using a random effects model, given the high likelihood of between-study variance due to differences in underlying population, as well as methodology. We pooled the point estimates from each study using the generic inverse-variance method of DerSimonian and Laird [12]. The heterogeneity of effect size estimates across these studies was quantified using the *I*^2^ statistic. An *I*^2^ value of 0–25% represented insignificant heterogeneity, 25–50% represented low heterogeneity, 50–75% represented moderate heterogeneity, and >75% represented high heterogeneity [13]. We also conducted a sensitivity analysis according to types of research studies (RCT vs. PSM). Meta-analysis using fixed effects models was also done. Funnel plots were constructed to evaluate publication biases and Egger's test. All statistical analyses were performed using RevMan software (version 5.4.1; Cochrane, London, United Kingdom) and Stata version 14 (College Station, Tx).

### 2.5. Risk of Bias

The risk of bias of randomized controlled trials (RCTs) was assessed using the Cochrane Collaboration risk assessment tool for RCTs [14]. Every study was assessed regarding the following domains: random sequence generation, allocation concealment, blinding of participants and personnel, blinding of outcome assessment, incomplete outcome data, selective reporting, and other biases. The quality of non-RCTs was assessed using the Newcastle–Ottawa Quality Scale (NOS) [15]. This scale assesses each study using three categories: (1) the representativeness of the subjects; (2) the comparability between the study groups; and (3) ascertainment of the exposure or outcome of interest, for case-control and cohort studies, respectively. Studies with total scores of >6 and <4 were considered to be of high and low quality, respectively.

## 3. Results

**Figure 1:**
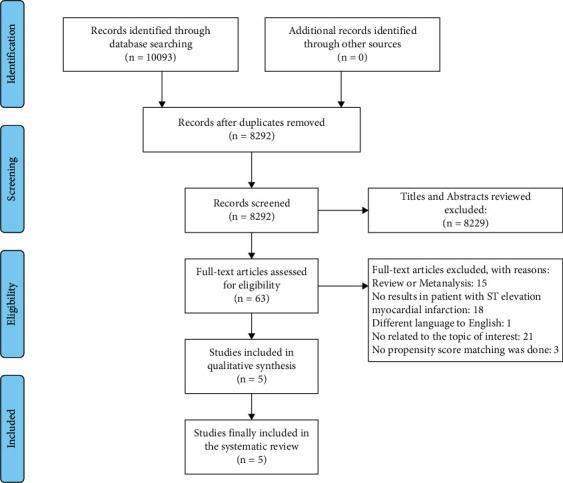
PRISMA flow chart. Selection process for studies to be included in the meta-analysis based on PRISMA standards.

We identified 10,093 potentially relevant studies. After removing duplicated and unsuitable studies by title and abstract review, 63 articles were considered potentially eligible and evaluated in depth. After full-text review, further 58 articles were rejected and 5 were included in the present meta-analysis.  Figure 1 summarizes the literature review process. All studies included were either randomized controlled trials or observational studies where PSM was done to match baseline characteristics.  Table 1 shows the main characteristics of these studies. The overall methodological quality of the studies included was generally good, as measured by the Cochrane collaboration tool for RCT and NOS for retrospective studies (Supplementary Table 1; Supplementary Figure 1).

**Figure 2:**
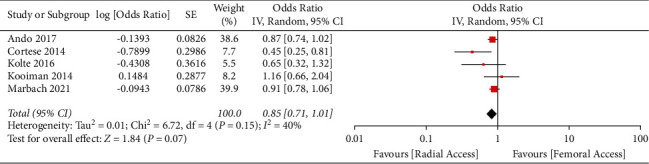
Forest plot. Forest plot demonstrating the no association of transradial access with lower risk of contrast induced acute kidney injury in patients undergoing primary percutaneous coronary intervention for ST-elevation myocardial infarction compared with transfemoral access.

**Figure 3:**
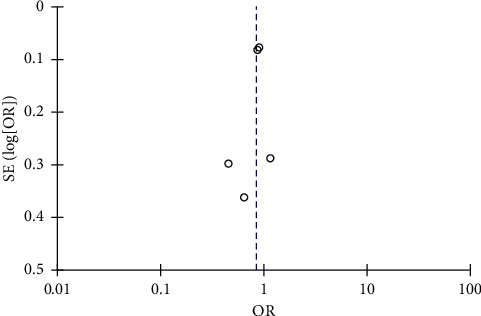
Funnel plot. Visual estimation of the funnel plot suggesting a minimal asymmetry, which was quantified to be statistically nonsignificant by means of Egger's regression test (*p*=0.409). Circles represent observed published studies.

**Figure 4:**
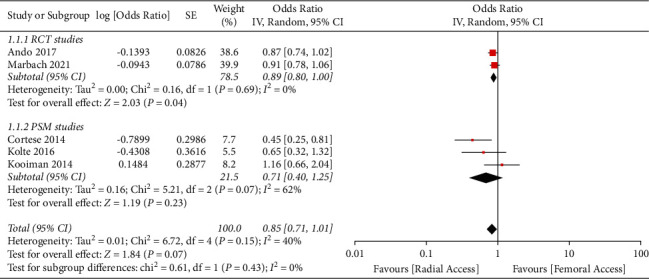
Forest plot. Forest plot using random effects model that demonstrates that transradial access is nonassociated with a significantly lower risk of contrast-induced acute kidney injury in patients undergoing primary percutaneous coronary intervention for ST-elevation myocardial infarction compared with transfemoral access even after dividing the studies by research type (group 1: randomized controlled trials, group 2: studies where propensity score matching was used).

A total of 5 multicenter studies (2 RCTs and 3 PSM studies) from 2014 to 2021 with a total of 8,536 STEMI patients were included [16–20]. Distribution of total TRA and TFA patient was almost even (4265 and 4271 for TRA and TFA, respectively). All studies were multicenter and defined AKI by either a relative increase of more than 25% from baseline or absolute increase of ≥0.5 mg/dl from baseline. However, the time-lapse of AKI was not coherent amongst the included studies. Mean age of all the studies population was >60 years, and the majority were males. Incidence of diabetes mellitus was reported in all the studies, ranging from 16.7% to 38.5% in the TRA and from 18.3% to 38.9% in the TFA. Presence of chronic kidney disease, congestive heart failure, and major bleeding events were not reported in most of the studies. All studies reported the mean contrast volume used, ranging from 183.3 ml to 227.6 ml in TRA and from 183.6 ml to 232.3 ml in TFA studies. All studies reported its respective adjusted odds ratio except the study by Cortese et al., where the odds ratio was calculated based on the incidence of AKI on each group. TRA was associated with numerically lower risk of AKI compared with TFA, but the effects were not statistically significant (odds ratio 0.85, 95% CI 0.71–1.01, p 0.07, I^2^ = 40%) (Figure 2). Visual estimation of the funnel plot (Figure 3) suggested a minimal asymmetry, which was quantified to be statistically nonsignificant by means of Egger's regression test (*p*=0.409). Sensitivity analysis divided by he type of research study was performed. It showed that TRA did not have a significant association with lower risk of AKI for both RCT and PSM group with odds ratio of 0.89 (95% CI 0.80–1.00, *I*^2^ = 0%) and 0.71 (95% CI 0.40–1.25, *I*^2^ = 62%), respectively (Figure 4) (Supplementary Figure 2). Meta-analysis using fixed effects models was also done, and it demonstrated that TRA was significantly associated with a reduced risk for AKI compared to TFA (odds ratio 0.87, 95% CI 0.79–0.97, *p* 0.01, *I*^2^ = 40%) (Supplementary Figures 3 and 4).

## 4. Discussion

In the meta-analysis using random effects models of RCT and PSM samples, TRA was not associated with a significantly lower risk of AKI in patients with STEMI undergoing primary PCI. However, in the metanalysis using fixed effects models, TRA was significantly associated with a lower risk of AKI compared to TFA.

Acute kidney injury is a known complication after primary PCI in patients with ACS. Its presence is associated with increased morbidity and mortality. The etiology is complex and multifactorial, with different mechanisms that interrelate and are active at different times and intensities [21]. The etiologies noted include the effect of the contrast media, which increases endothelin and adenosine and reduces the availability of nitric oxide, leading to vasoconstriction and hypoxia of the renal outer medulla. Next includes cholesterol embolization, where cholesterol crystals flow to the distal vessels of the bloodstream, blocking renal arterioles and causing multiple renal microembolisms. Procedure-related factors such as bleeding complications have also been described, such as in the case of hypoperfusion, or the need of transfusing subsequent heme products to replace losses, that can end up causing an impairment of tissue oxygen delivery and predisposition to inflammatory response and oxidative stress [22]. And finally, the presence of cardiac insufficiency leads to a decrease in renal blood flow and a consequent deterioration of the glomerular filtration rate. The deleterious effect acute cardiac insufficiency on renal function is also known as type-I cardiorenal syndrome [23].

Even though access site choice for primary PCI in patients with ACS per se is not expected to influence the development of AKI, potential reasons that could explain why the access site contributes to the development of AKI is thought to be mainly due to cholesterol embolization or procedure-related complications, as described in the most recent literature [17]. Cholesterol crystals embolize into the renal arteries during catheter manipulation in the aorta, and due to less contact of catheters to the aortic wall, it may be less common in PCI undergone by TRA [24]. Bleeding and transfusions are more common in TFA [22]; furthermore, current data suggest that significant periprocedural bleeding events, particularly the need for blood transfusion, may be the most important determinants of AKI following primary PCI in STEMI patients [18]. Therefore, in cases where access site contributes to bleeding events, there is a potential for an increased AKI incidence.

Studies have shown risk reductions of major bleeding events, mortality, and vascular complications when using TRA site compared with TFA in patients with STEMI [25]. Transradial access has also shown to reduce the risk of major adverse cardiac events, days of hospital stay, stroke, atheroma, and embolization in patients with STEMI [26, 27]. Interestingly, Mehta et al. demonstrated that there was a reduced risk of death, stroke, and acute myocardial infarction when using TRA over TFA in patients with STEMI; however, there was no such benefit observed in patients with non-STEMI [28]. This brings concern and doubt if a similar finding occurs in the development of AKI.

As demonstrated in the metanalyses by Ando et al. and Wang et al. [7–9], when adult patients require coronary catheterization for any reason (PCI or coronary angiography) irrespective of the presence of any type of ACS or not, TRA is associated with a reduced risk of AKI compared to TFA. On the contrary, however, our metanalysis demonstrated that in cases where primary PCI is done in the presence of STEMI, TRA does not confer a significant lower risk of AKI.

To elucidate the risk difference between the access sites with AKI in patients with STEMI, studies have been performed in selected populations [16–20]. Kooiman et al. demonstrated a reduced incidence of AKI in patients with TRA access over TFA access. In spite of this, after the propensity matching, a significant difference amongst TRA and TFA access in STEMI patients was not observed [17]. In 2016, Kolte et al. published a study of 508 patients that had a mild reduction in risk of AKI in patients with TRA access PCI, but this difference was not statistically significant. In their study, they described there was a reduced risk of AKI in TRA coronary intervention over TFA access with the following factors: lower rates of bleeding and avoidance of catheter contact with descending aorta. Consequently, these factors reduce the risk of a cholesterol embolization to renal arteries [20]. Cortese et al. compared AKI in patients with differing PCI access sites and found that, in this 450-patient cohort, there were lower rates of AKI in patients with TRA. Long-term follow-up has shown that TRA access has a reduced risk of chronic kidney disease over TFA access [16]. The limitations of this study were as follows: follow-up was limited to hospital stay and there was no exact information of how many patients were treated with N-acetylcysteine or bicarbonate infusion for AKI. The AKI-SAFARI [18] and the STEMI subgroup in the AKI-MATRIX [19] trials demonstrated no association between catheterization access site and AKI, irrespective of the AKI definition applied. It should also be noted that the insignificant differences in bleeding and transfusion requirements made between the two groups (TFA and TRA) were what lead to the nonstatistical difference in AKI. However, instead of focusing solely on access sites as a strategy to reduce AKI in patients undergoing PCI, the focus should be on avoiding other potential causes that can lead to bleeding complications [29]. These include the use of smaller size sheaths, fluoroscopic landmarking, ultrasound guidance, and considerations of vascular closure devices.

The main strength of this study is that it is the first meta-analysis to evaluate the association of vascular access and the development of AKI in patients with STEMI, and it is important to note that all the studies included were multicenter RCT and observational studies where PSM was done to match the baseline characteristics.

On the other hand, our meta-analysis has several potential limitations. First, as any other meta-analysis, there is a publication bias. For example, studies that show a neutral outcome in mortality are less likely to be published than those that show a positive outcome and thus tend to bias towards a more positive outcome. Second, the two RCT studies contributed to almost 91% (Marbach et al. and Ando et al.) of all the patients analyzed as part of this meta-analysis. Hence, it is possible that their data sets may have driven the outcome of the pooled meta-analysis. But, sensitivity analyses where RCTs were analyzed separately from the rest of the studies demonstrated that the results were the same. Third, the metanalysis using the fixed effects model demonstrated that TRA was significantly associated with a reduced risk of AKI compared to TFA unlike what was demonstrated by the random effects model. This can bring doubts as to what the interpretation should be. Thus, we described the results in the two metanalysis analyses and added the figures in the supplementary material. In the end, we used the results given by the random effects model because there is a high likelihood that the between-study variance was due to differences in underlying population, as well as methodology. Lastly, there were different time frames used in the evaluations of AKI across the studies.

## 5. Conclusion

We found that TRA was not significantly associated with a lower risk of AKI in patients undergoing primary PCI for STEMI compared with TFA, contrary to what has been shown in previous studies which have stated that TRA is protective. Rather than focusing on access site by itself as a strategy to reduce AKI in patients with STEMI undergoing PCI, a larger focus should be placed on bleeding prevention. There should be additional rigorous studies to clarify this outcome.

## Figures and Tables

**Table 1:** Characteristics of included studies.

Author	Year	Study type	Number of centers	Total population	Total patients with TRA	Total patients with TFA	AKI definition	AKI time lapse	Age mean (SD)		Male sex (%)		Diabetes (%)		CKD (%)		GFR (ml/min/1.73 m^2^)		Congestive heart failure (%)		Mean LVEF (%) (SD)		Major bleeding (%)		Mean contrast volume ml (SD)		Incidence of AKI (*n*)	
									TRA	TFA	TRA	TFA	TRA	TFA	TRA	TFA	TRA	TFA	TRA	TFA	TRA	TFA	TRA	TFA	TRA	TFA	TRA	TFA
Cortese	2014	RPSM	4	450	225	225	>0.5 mg/dL or >25%	Highest within 3 days	64.4 (12.5)	64.3 (12.7)	76.40	75.2	22	22	NA	NA	NA	NA	NA	NA	47.3 (9.4)	47.3 (9.4)	NA	NA	193.3 (64.0)	188.3 (69.3)	19	38
Kooiman	2014	RPSM	47	1341	677	664	>0.5 mg/dL	Highest within 7 days	63.9 (11.6)^*∗*^	63.8 (12.1)^*∗*^	69.5^*∗*^	69.6^*∗*^	38.5^*∗*^	38.9^*∗*^	66.6^*∗*^	66.4^*∗*^	81.6 (27.9)^*∗*^	81.2 (27.2)^*∗*^	14.1^*∗*^	15.3^*∗*^	54.1 (11.8)^*∗*^	52.9 (12)^*∗*^	0.8^*∗*^^$^	2.9^*∗*^^$^	189.4 (78.1)^*∗*^	191.7 (78.1)^*∗*^	127^*∗*^	172^*∗*^
Kolte	2016	RPSM	2	508	254	254	>0.5 mg/dL or > 25%	Highest within 3 days	60.1 (12.1)	60.6 (12)	70.7	71.1	22	24	2	2	NA	NA	NA	NA	50 (10.4)	50.1 (10.3)	NA	NA	186.1 (63.5)	183.6 (73.5)	14	21
Ando	2017	RCT	78	3952	1977	1975	>0.5 mg/dL or > 25%	Highest during hospitalization	65.5 (11.8)^	65.9 (11.8)^	74.5^	72.6^	22.8^	22.4^	NA	NA	84.2 (25.3)^	83.4 (25.5)^	8.6^	9.2^	NA	NA	NA	NA	183.3 (104.5)^	183.9 (110.1)^	354	397
Marbach	2021	RCT	5	2285	1132	1153	>0.5 mg/dL or > 25%	Highest within 3 days	61.6 (12.2)	61.9 (12.1)	77.8	78.1	16.7	18.3	NA	NA	86.1 (26.9)	86.7 (26.5)	NA	NA	NA	NA	1.6^&^	2.3^&^	227.6 (76.7)	232.3 (73.5)	243	226

TRA: transradial access, TFA: transfemoral access, AKI: acute kidney injury, SD: standard deviation, CKD: chronic kidney disease, GFR: glomerular filtration rate, LVEF: left ventricular ejection fraction, RPSM: retrospective propensity score matching sample, and RCT: randomized controlled trial. NA: not available. ^∗^From the propensity score matching data, no STEMI exclusively. ^From the total population, no STEMI. ^$^Postprocedural bleeding was defined as bleeding within 72 hours after PCI causing a drop in hematocrit >10% and a drop in hemoglobin levels ≥ 3 g/dL, or requiring transfusion of ≥ 1 unit of whole blood. ^&^Bleeding defined based on TIMI bleeding criteria (any major or minor).

## Data Availability

The data used to support the findings of this study are available from the corresponding author upon request.

## Supplementary Materials

Supplementary Materials (1) Search Strategy. (2) Risk of bias assessment. (2.1) *Supplementary Table 1*. Risk of bias assessment for nonrandomized studies using Newcastle–Ottawa scale. (2.2) *Supplemental Figure 1*. Risk of bias of included randomized controlled trials using the Cochrane Collaboration Risk Assessment tool, demonstrating that the quality was generally good. (3) Sensitivity analysis. (3.1) *Supplementary Figure 2*. Funnel plot subgroups random effects model. (3.2) *Supplementary Figure 3*. Forest Plot. (3.3) *Supplementary Figure 4*. Funnel plot fixed effects model.
