# Supplementary material for: Transradial versus Transfemoral Access and the Risk of Acute Kidney Injury following Primary Percutaneous Coronary Intervention in Patients with ST-Elevation Myocardial Infarction: A Systematic Review and Meta-Analysis of Randomized Controlled Trials and Propensity-Score-Matched Studies
Source: J Interv Cardiol. 2022 Mar 10;2022:6774439. doi: 10.1155/2022/6774439 (PMC8930211; doi:10.1155/2022/6774439)
Supplement: Supplementary Materials — (1) Search Strategy. (2) Risk of bias assessment. (2.1) Supplementary Table 1. Risk of bias assessment for nonrandomized studies using Newcastle–Ottawa scale. (2.2) Supplemental Figure 1. Risk of bias of included randomized controlled trials using the Cochrane Collaboration Risk Assessment tool, demonstrating that the quality was generally good. (3) Sensitivity analysis. (3.1) Supplementary Figure 2. Funnel plot subgroups random effects model. (3.2) Supplementary Figure 3. Forest Plot. (3.3) Supplementary Figure 4. Funnel plot fixed effects model. [file 6774439.f1.zip › 6774439.f1/Appendix_Radial_Femoral_0125.docx]

Supplementary Material.

1. **Search strategy:**

Total papers found in Pubmed: 5693

Search: **((((((((((((radial) OR (transradial)) AND (femoral)) OR (transfemoral)) AND (coronary angiography)) OR (percutaneous coronary intervention)) OR (left heart catheterization)) AND (st elevation myocardial infarction)) OR (STEMI)) AND (acute kidney injury)) OR (contrast induced nephropathy)) NOT (child)) NOT (animals)** Sort by: **Most Recent**

(((((((((("radial artery"[MeSH Terms] OR ("radial"[All Fields] AND "artery"[All Fields]) OR "radial artery"[All Fields] OR "radial"[All Fields] OR "radially"[All Fields] OR "radials"[All Fields] OR ("transradial"[All Fields] OR "transradially"[All Fields])) AND ("femor"[All Fields] OR "femorals"[All Fields] OR "femur"[MeSH Terms] OR "femur"[All Fields] OR "femoral"[All Fields])) OR ("transfemoral"[All Fields] OR "transfemorally"[All Fields])) AND ("coronary angiography"[MeSH Terms] OR ("coronary"[All Fields] AND "angiography"[All Fields]) OR "coronary angiography"[All Fields])) OR ("percutaneous coronary intervention"[MeSH Terms] OR ("percutaneous"[All Fields] AND "coronary"[All Fields] AND "intervention"[All Fields]) OR "percutaneous coronary intervention"[All Fields]) OR ("left"[All Fields] AND ("heart catheterisation"[All Fields] OR "cardiac catheterization"[MeSH Terms] OR ("cardiac"[All Fields] AND "catheterization"[All Fields]) OR "cardiac catheterization"[All Fields] OR ("heart"[All Fields] AND "catheterization"[All Fields]) OR "heart catheterization"[All Fields]))) AND ("st elevation myocardial infarction"[MeSH Terms] OR ("st"[All Fields] AND "elevation"[All Fields] AND "myocardial"[All Fields] AND "infarction"[All Fields]) OR "st elevation myocardial infarction"[All Fields])) OR ("st elevation myocardial infarction"[MeSH Terms] OR ("st"[All Fields] AND "elevation"[All Fields] AND "myocardial"[All Fields] AND "infarction"[All Fields]) OR "st elevation myocardial infarction"[All Fields] OR "stemi"[All Fields] OR "stemis"[All Fields])) AND ("acute kidney injury"[MeSH Terms] OR ("acute"[All Fields] AND "kidney"[All Fields] AND "injury"[All Fields]) OR "acute kidney injury"[All Fields])) OR (("contrast media"[Pharmacological Action] OR "contrast media"[MeSH Terms] OR ("contrast"[All Fields] AND "media"[All Fields]) OR "contrast media"[All Fields] OR "contrast"[All Fields] OR "contrasted"[All Fields] OR "contrasting"[All Fields] OR "contrastive"[All Fields] OR "contrastively"[All Fields] OR "contrastiveness"[All Fields] OR "contrastivity"[All Fields] OR "contrasts"[All Fields]) AND ("induce"[All Fields] OR "induced"[All Fields] OR "inducer"[All Fields] OR "inducers"[All Fields] OR "induces"[All Fields] OR "inducibilities"[All Fields] OR "inducibility"[All Fields] OR "inducible"[All Fields] OR "inducing"[All Fields]) AND ("kidney diseases"[MeSH Terms] OR ("kidney"[All Fields] AND "diseases"[All Fields]) OR "kidney diseases"[All Fields] OR "nephropathies"[All Fields] OR "nephropathy"[All Fields]))) NOT ("child"[MeSH Terms] OR "child"[All Fields] OR "children"[All Fields] OR "child s"[All Fields] OR "children s"[All Fields] OR "childrens"[All Fields] OR "childs"[All Fields])) NOT ("animals"[MeSH Terms:noexp] OR "animals"[All Fields])

**Translations**

**radial:** "radial artery"[MeSH Terms] OR ("radial"[All Fields] AND "artery"[All Fields]) OR "radial artery"[All Fields] OR "radial"[All Fields] OR "radially"[All Fields] OR "radials"[All Fields]

**transradial:** "transradial"[All Fields] OR "transradially"[All Fields]

**femoral:** "femor"[All Fields] OR "femorals"[All Fields] OR "femur"[MeSH Terms] OR "femur"[All Fields] OR "femoral"[All Fields]

**transfemoral:** "transfemoral"[All Fields] OR "transfemorally"[All Fields]

**coronary angiography:** "coronary angiography"[MeSH Terms] OR ("coronary"[All Fields] AND "angiography"[All Fields]) OR "coronary angiography"[All Fields]

**percutaneous coronary intervention:** "percutaneous coronary intervention"[MeSH Terms] OR ("percutaneous"[All Fields] AND "coronary"[All Fields] AND "intervention"[All Fields]) OR "percutaneous coronary intervention"[All Fields]

**heart catheterization:** "heart catheterisation"[All Fields] OR "cardiac catheterization"[MeSH Terms] OR ("cardiac"[All Fields] AND "catheterization"[All Fields]) OR "cardiac catheterization"[All Fields] OR ("heart"[All Fields] AND "catheterization"[All Fields]) OR "heart catheterization"[All Fields]

**st elevation myocardial infarction:** "st elevation myocardial infarction"[MeSH Terms] OR ("st"[All Fields] AND "elevation"[All Fields] AND "myocardial"[All Fields] AND "infarction"[All Fields]) OR "st elevation myocardial infarction"[All Fields]

**STEMI:** "st elevation myocardial infarction"[MeSH Terms] OR ("st"[All Fields] AND "elevation"[All Fields] AND "myocardial"[All Fields] AND "infarction"[All Fields]) OR "st elevation myocardial infarction"[All Fields] OR "stemi"[All Fields] OR "stemis"[All Fields]

**acute kidney injury:** "acute kidney injury"[MeSH Terms] OR ("acute"[All Fields] AND "kidney"[All Fields] AND "injury"[All Fields]) OR "acute kidney injury"[All Fields]

**contrast:** "contrast media"[Pharmacological Action] OR "contrast media"[MeSH Terms] OR ("contrast"[All Fields] AND "media"[All Fields]) OR "contrast media"[All Fields] OR "contrast"[All Fields] OR "contrasted"[All Fields] OR "contrasting"[All Fields] OR "contrastive"[All Fields] OR "contrastively"[All Fields] OR "contrastiveness"[All Fields] OR "contrastivity"[All Fields] OR "contrasts"[All Fields]

**induced:** "induce"[All Fields] OR "induced"[All Fields] OR "inducer"[All Fields] OR "inducers"[All Fields] OR "induces"[All Fields] OR "inducibilities"[All Fields] OR "inducibility"[All Fields] OR "inducible"[All Fields] OR "inducing"[All Fields]

**nephropathy:** "kidney diseases"[MeSH Terms] OR ("kidney"[All Fields] AND "diseases"[All Fields]) OR "kidney diseases"[All Fields] OR "nephropathies"[All Fields] OR "nephropathy"[All Fields]

**child:** "child"[MeSH Terms] OR "child"[All Fields] OR "children"[All Fields] OR "child's"[All Fields] OR "children's"[All Fields] OR "childrens"[All Fields] OR "childs"[All Fields]

**animals:** "animals"[MeSH Terms:noexp] OR animals[All Fields]

Total papers found in Embase: 4086.

(((((**radial**:ti,ab,kw OR **transradial**:ti,ab,kw) AND **femoral**:ti,ab,kw OR **transfemoral**:ti,ab,kw) AND **'coronary angiography'**:ti,ab,kw OR **'percutaneous coronary intervention'**:ti,ab,kw OR **'left heart catheterization'**:ti,ab,kw) AND **'st elevation myocardial infarction'**:ti,ab,kw OR **stemi**:ti,ab,kw) AND **'acute kidney injury'**:ti,ab,kw OR **'contrast induced nephropathy'**:ti,ab,kw) NOT **child** NOT **animal**

Total papers found in Scopus: 314

( ALL ( radial )  OR  ALL ( transradial )  AND  ALL ( femoral )  OR  ALL ( transfemoral )  AND  ALL ( coronary  AND angiography )  OR  ALL ( percutaneous  AND coronary  AND intervention )  OR  ALL ( left  AND heart  AND catheterization )  AND  ALL ( st  AND elevation  AND myocardial  AND infarction )  OR  ALL ( stemi )  AND  ALL ( acute  AND kidney  AND injury )  OR  ALL ( contrast  AND induced  AND nephropathy )  AND NOT  ALL ( child )  AND NOT  ALL ( animal ) )

1. **Risk of Bias assessment:**
   1. **Supplementary Table 1.** Risk of Bias Assessment for non-randomized studies using Newcastle Ottawa scale:

| **The Newcastle-Ottawa Scale (NOS) for assessing the quality of nonrandomized studies in meta-analysis** | | | | |
| --- | --- | --- | --- | --- |
|  |  |  |  |  |
| **Observational** | **Selection** | **Comparability** | **Outcome/Exposure** | **Total** |
| Cortese et al ,2014 | *** | ** | * | 6 |
| Kolte et al,2016 | *** | ** | * | 6 |
| Kooiman et al,2014 | *** | ** | *** | 8 |

- 1. Risk of bias of included randomized controlled trials using the Cochrane Collaboration risk assessment tool

**Supplemental Figure 1.** Risk of bias of included randomized controlled trials using the Cochrane Collaboration risk assessment tool, demonstrating that the quality was generally good.


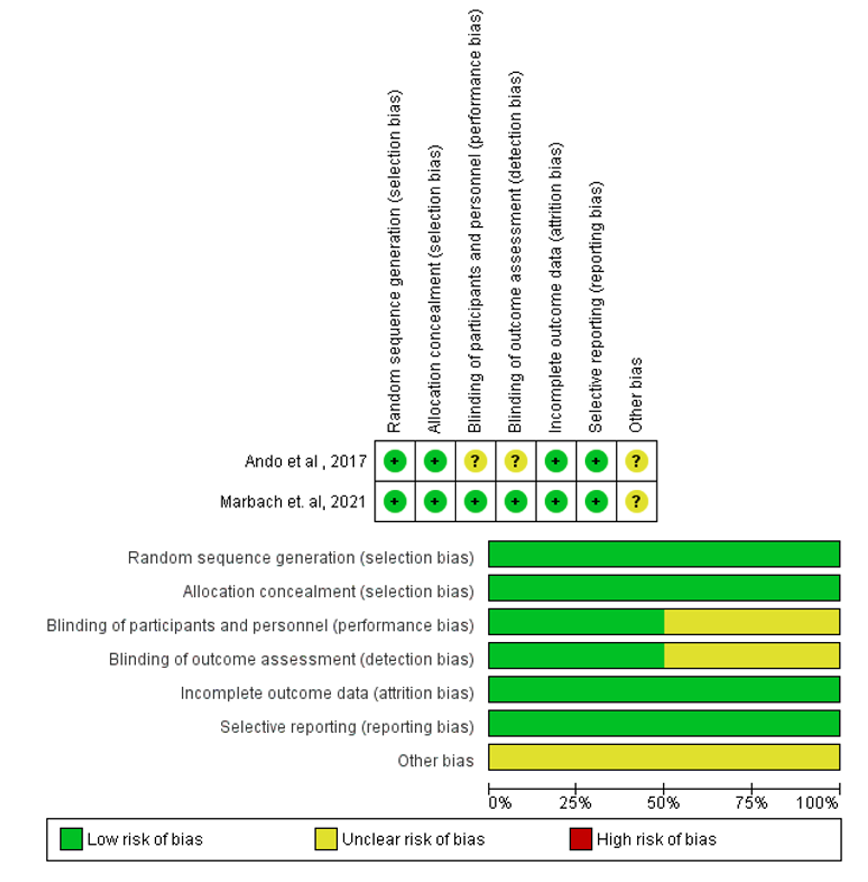


1. **Sensitivity Analysis**

**Supplementary Figure 2. Funnel Plot Subgroups Random Effects Model**


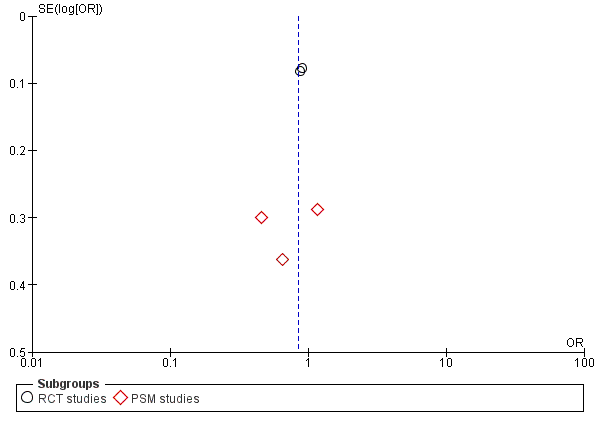


**Supplementary Figure 3. Forest Plot.**

Forest plot using fixed effects model that demonstrates that transradial access is associated to a significant lower risk of contrast induced acute kidney injury in patients undergoing primary percutaneous coronary intervention for st-elevation myocardial infarction compared with transfemoral access.


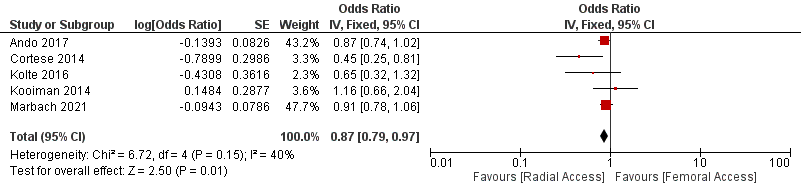


**Supplementary Figure 4. Funnel Plot Fixed Effects Model.**

**
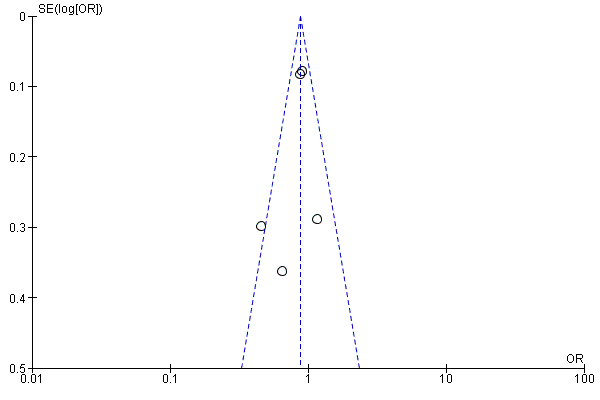
**
