# Supplementary figures and images for: Transradial versus Transfemoral Access and the Risk of Acute Kidney Injury following Primary Percutaneous Coronary Intervention in Patients with ST-Elevation Myocardial Infarction: A Systematic Review and Meta-Analysis of Randomized Controlled Trials and Propensity-Score-Matched Studies
Source: J Interv Cardiol. 2022 Mar 10;2022:6774439. doi: 10.1155/2022/6774439 (PMC8930211; doi:10.1155/2022/6774439)

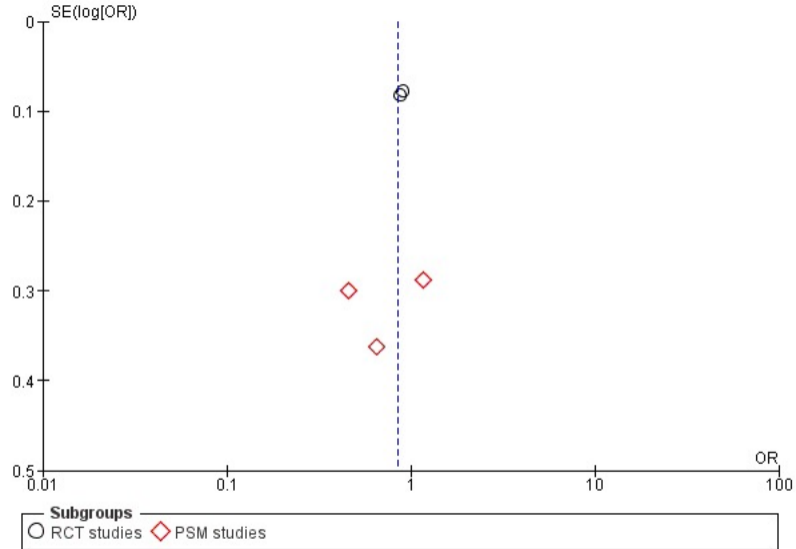

Supplement: Supplementary Materials — (1) Search Strategy. (2) Risk of bias assessment. (2.1) Supplementary Table 1. Risk of bias assessment for nonrandomized studies using Newcastle–Ottawa scale. (2.2) Supplemental Figure 1. Risk of bias of included randomized controlled trials using the Cochrane Collaboration Risk Assessment tool, demonstrating that the quality was generally good. (3) Sensitivity analysis. (3.1) Supplementary Figure 2. Funnel plot subgroups random effects model. (3.2) Supplementary Figure 3. Forest Plot. (3.3) Supplementary Figure 4. Funnel plot fixed effects model. [file 6774439.f1.zip › 6774439.f1/Supplementary Figure2 (1).pdf]

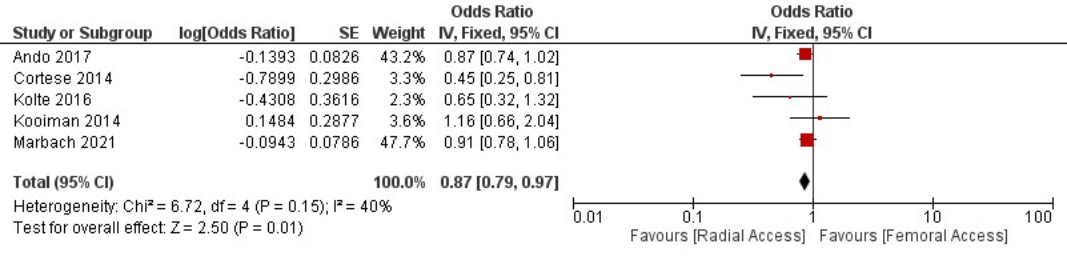

Supplement: Supplementary Materials — (1) Search Strategy. (2) Risk of bias assessment. (2.1) Supplementary Table 1. Risk of bias assessment for nonrandomized studies using Newcastle–Ottawa scale. (2.2) Supplemental Figure 1. Risk of bias of included randomized controlled trials using the Cochrane Collaboration Risk Assessment tool, demonstrating that the quality was generally good. (3) Sensitivity analysis. (3.1) Supplementary Figure 2. Funnel plot subgroups random effects model. (3.2) Supplementary Figure 3. Forest Plot. (3.3) Supplementary Figure 4. Funnel plot fixed effects model. [file 6774439.f1.zip › 6774439.f1/Supplementary Figure3 (1).pdf]

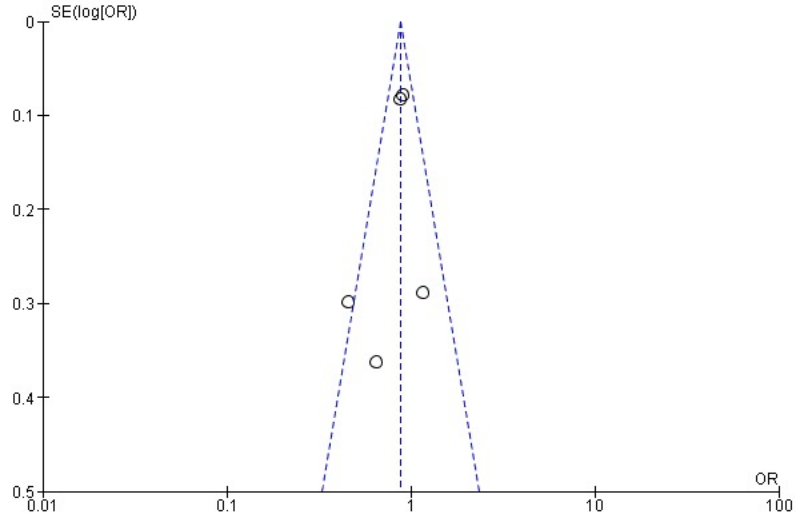

Supplement: Supplementary Materials — (1) Search Strategy. (2) Risk of bias assessment. (2.1) Supplementary Table 1. Risk of bias assessment for nonrandomized studies using Newcastle–Ottawa scale. (2.2) Supplemental Figure 1. Risk of bias of included randomized controlled trials using the Cochrane Collaboration Risk Assessment tool, demonstrating that the quality was generally good. (3) Sensitivity analysis. (3.1) Supplementary Figure 2. Funnel plot subgroups random effects model. (3.2) Supplementary Figure 3. Forest Plot. (3.3) Supplementary Figure 4. Funnel plot fixed effects model. [file 6774439.f1.zip › 6774439.f1/Supplementray Figure4 (1).pdf]
